# Exploring the relationship between psychosocial factors, work engagement, and mental health: a structural equation modeling analysis among faculty in Saudi Arabia

**DOI:** 10.1186/s12889-024-19114-4

**Published:** 2024-06-24

**Authors:** Nawal Ayyashi, Amira Alshowkan, Emad Shdaifat

**Affiliations:** 1 Master of Psychiatric and Mental Health Nursing, College of Nursing, Imam Abdulrahman Bin Faisal University, Dammam, Saudi Arabia; 2 Community Nursing Department, College of Nursing, Imam Abdulrahman Bin Faisal University, P.O Box 1982, Dammam, Saudi Arabia

**Keywords:** Psychosocial factors, Work engagement, Mental health, Structural equation modeling, Faculty, Saudi Arabia

## Abstract

**Background:**

Psychosocial hazards in the workplace were identified as a considerable risk to employee mental health as well as their general well-being. Few studies were found to examine its relationship with work engagement and mental health. Thus, this study examines the relationships between psychosocial factors, work engagement, and mental health within the faculty in Saudi Arabia using structural equation modeling.

**Methods:**

The cross-sectional study was conducted with a sample size of 375 faculty. Data collection was done using a self-administered online survey that included instruments such as the Copenhagen Psychosocial Questionnaire (COPSOQ), Utrecht Work Engagement Scale (UWES), and General Health Questionnaire (GHQ-12). SmartPLS 3 software facilitated data analysis and included the assessment of factors. Structural equation modelling was used to examine the interplay between psychosocial factors, work engagement, and mental health.

**Results:**

The robust measurement model was characterized by high loadings (0.719 to 0.970), Cronbach’s alpha (0.595 to 0.933), and composite reliability (0.807 to 0.968). Convergent and discriminant validity were confirmed using AVE and various criteria. The fit of the saturated model was superior. Burnout explained significant variance (0.585) with predictive relevance for all constructs. Notably, the impact of burnout on family conflict and the influence of stress on burnout were found to have significant effect sizes.

**Conclusion:**

The study uses structural equation modeling to examine the relationships between psychosocial factors, work engagement, and mental health among faculty in Saudi Arabia. The robust measurement model demonstrated high reliability and validity, while the saturated model demonstrated excellent fit. These findings contribute to our understanding of psychosocial dynamics, work engagement, and overall health among faculty in Saudi Arabia.

## Background

Psychosocial risks at work are components of work design as well as the social, organizational, and managerial settings of work that have the potential to inflict psychological or physical damage [1]. Among the most difficult concerns in occupational safety and health are work-related stress and psychosocial risks, which have a considerable influence on the health of people, organizations, and national economies [2].

Workplace psychosocial risks are serious threats to workers’ emotional and physical well-being. These dangers could lead to numerous health issues and work-related mishaps [3]. Low job satisfaction, health problems, accidents at work, stress at work, and burnout are all associated with psychosocial risks [1]. Depression and anxiety are more common, and there is a correlation between work-related stress and a decline in social contact and concentration on the job, as well as an increase in physical pain and cardiovascular problems. Anxiety at work is associated with several psychosocial risks [4–7].

The Job Demands-Resources (JD-R) Model provides a comprehensive framework for comprehending the connection between work characteristics and the well-being of employees. Lack of job management, organization, and social environments can lead to stress, despair, and other psychological, physiological, and social consequences. Psychosocial risks encompass several factors, such as organizational culture, workplace function, stress levels, work pace, and work relationships, that can impact performance and well-being in the workplace [8]. A person’s emotional control, adaptability, and a healthy mind-body relationship are aspects that contribute to mental well-being, a constantly changing internal state of balance essential for maximizing their abilities in line with societal standards [9].

The JD-R model suggests that occupational demands, like an intense workload or emotional stress, can deplete an employee’s energy reserves. Over time, this could lead to health problems like burnout. Employees demonstrate higher levels of engagement and commitment to their work when they have more freedom, receive constructive feedback, and receive social support from their employers. A study conducted further supports this notion, suggesting that employees who are aware of the resources available to them in their profession are more willing to invest additional time and effort in their work [10, 11]. This highlights the importance of providing employees with the necessary tools and support to thrive in their roles.

Studies estimate that 17.6% of the working population experiences mental health challenges annually, impacting work capacity and performance [12]. With the third Sustainable Development Goal (SDG) focusing on global health and well-being, the role of education, particularly the psychosocial health of educators, becomes paramount [13]. An emotional, cognitive, and psychological construct that refers to a good and satisfying mental state associated with work, engagement is an emotional, cognitive, and psychological construct that is founded on three dimensions: vigor, devotion, and absorption [3].

In this context, job engagement, characterized by vitality, commitment, and absorption, emerges as a positive mental condition associated with work [14]. However, the teaching profession, known for its high occupational stress, often leaves educators feeling overwhelmed and stressed [15, 16]. University lecturers, in addition to teaching and advising, contend with administrative tasks, research, family responsibilities, and social commitments, contributing to elevated stress levels [17]. High job engagement is correlated with lower psychosocial hazards and burnout, highlighting the significance of addressing mental health concerns for optimal job performance [18].

Despite the existing research on workplace stress, psychosocial risks, and mental health, there is a notable gap in understanding these issues, specifically among faculty members in Saudi Arabia. This study aims to fill this gap by exploring the relationship between work engagement, psychosocial risks, and mental health among faculty members in Saudi Arabia, ultimately contributing essential insights for the development of effective strategies to enhance the well-being and engagement of academic staff. The main purpose of the present study was to examine the complex relationships between psychosocial factors, work engagement, and mental health within the faculty in Saudi Arabia using structural equation modeling.

The aim is to examine the complex relationships between psychosocial factors, work engagement, and mental health within the faculty in Saudi Arabia using structural equation modelling.

## Hypotheses

**Fig. 1:**
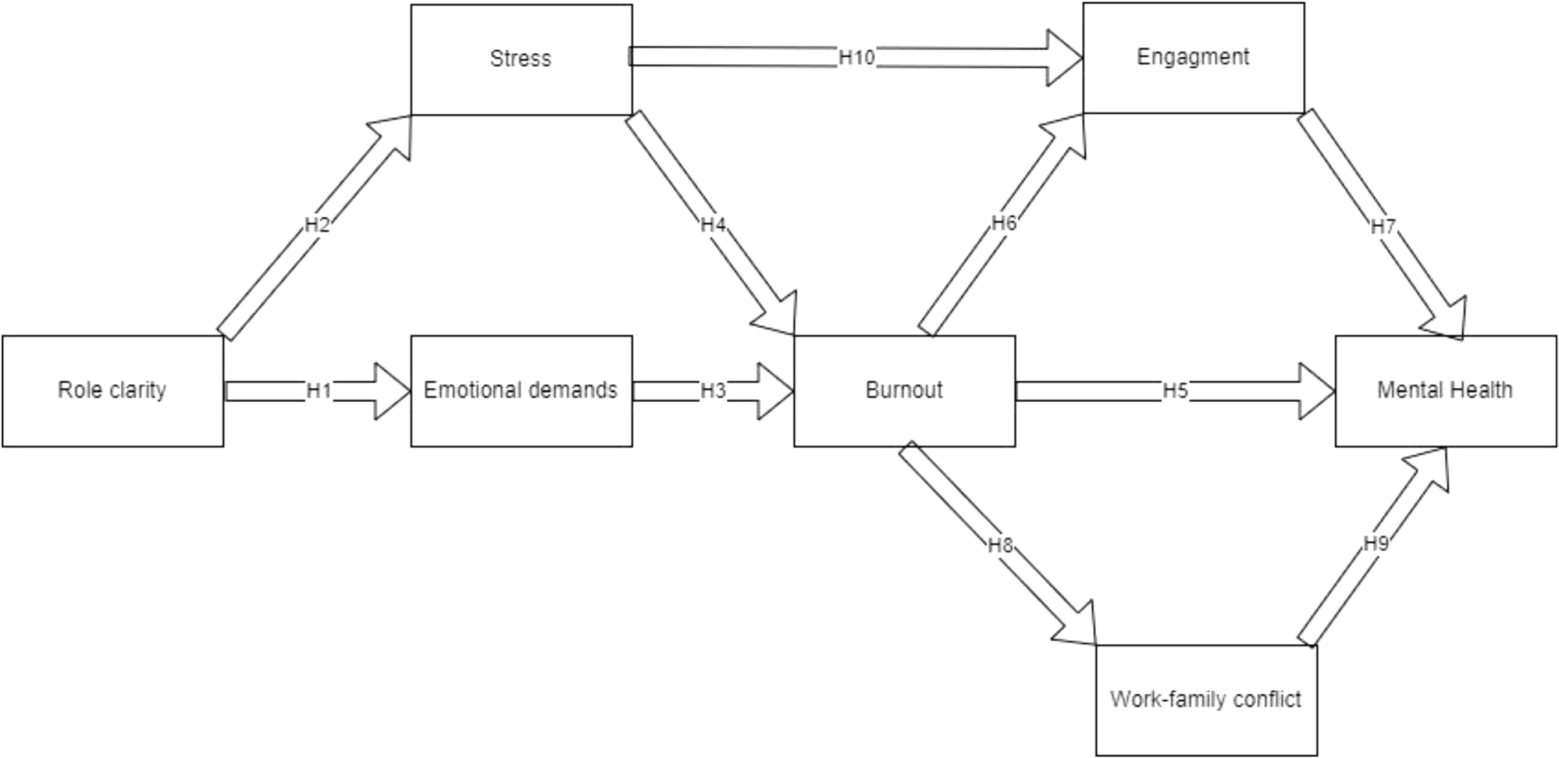
Conceptual Framework

Based on the conceptual framework (Fig. 1), the following are the study’s hypotheses:

- H1: There is a relationship between role clarity and emotional demand.
- H2: There is a relationship between role clarity and stress.
- H3: There is a relationship between emotional demand and burnout.
- H4: There is a relationship between stress and burnout.
- H5: There is a relationship between burnout and mental health.
- H6: There is a relationship between burnout and engagement.
- H7: There is a relationship between engagement and mental health.
- H8: There is a relationship between burnout and work-family conflict.
- H9: There is a relationship between work-family conflict and mental health.
- H10: There is a relationship between stress and engagement.

## Methods

### Site, setting, and design

This cross-sectional study was conducted in the Eastern region of Saudi Arabia and specifically targeted data collection from faculty members working at Imam Abdulrahman Bin Faisal University. It was an integral part of a research project aimed at assessing the relationships between psychosocial factors, work engagement, and mental health in an academic context in Saudi Arabia.

### Sample size and calculation

The sample size for estimating proportion was using the Stephen Thompson formula [19] based on the total academic staff of Imam Abdulrahman Bin Faisal University, the sample size (*n*) of the study was calculated as follows:

$${\rm{n = Np}}\left( {{\rm{1 - p}}} \right){\rm{ \div [}}\left( {{\rm{N - 1}}} \right)\left( {{{{{\rm{d}}^{\rm{2}}}} \over {{{\rm{z}}^{\rm{2}}}}}} \right){\rm{ + p}}\left( {{\rm{1 - p}}} \right){\rm{]}}$$

The total number of faculty members (N) was 2412. The percentage error (d) was set at 0.05, and the estimated proportion of the population with a certain characteristic (p) was 0.50. The upper α/2 point of the normal distribution (z) was 1.96. A total of 375 faculty members from various colleges of Imam Abdulrahman Bin Faisal University. Participants were randomly selected, and the inclusion criteria required at least one year of university-level teaching experience. In this context, *p* is the estimated proportion of the population with a particular attribute, *d* is the margin of error (expressed as a proportion), and *z* is the Z-score corresponding to the desired confidence level. The numerator Np(1-p) represents the variance of the population, While the denominator term (N-1)(d2/z2) + p(1-p) incorporates finite population correction.

## Data collection tools

Data collection involved the administration of a self-administered online survey, employing three distinct instruments. Firstly, a section addressing demographic factors was included to explore various elements influencing participants’ profiles. The second instrument utilized was the Copenhagen Psychosocial Questionnaire (COPSOQ) by [20], designed to evaluate psychosocial risks across dimensions such as work demands, organizational factors, interpersonal connections, and health. Comprising 23 dimensions and 40 questions, each answered on a 5-point Likert scale, COPSOQ’s reliability is notably high, with an internal consistency measured by Cronbach’s alpha of 0.92.

The third instrument employed was the Utrecht Work Engagement Scale (UWES) by [21], assessing work engagement through the dimensions of vigor, dedication, and absorption. This scale, consisting of nine questions, utilized a 7-point Likert scale for responses. The reliability indices, expressed as Cronbach’s alpha, were 0.82 for vigor, 0.86 for dedication, and 0.80 for absorption. Finally, the General Health Questionnaire [GHQ-12] by [22] constituted the fourth instrument, comprising twelve items to evaluate mental health, utilizing Likert ratings ranging from 0 to 3. This questionnaire incorporated both positive and negative phrasing, with the GHQ-12 demonstrating robust reliability in various studies, featuring Cronbach alphas ranging from 0.76 to 0.86 within the Spanish population.

### Ethical consideration

This study was based on strict ethical considerations. Before participation, participants were provided with a comprehensive informed consent document explaining the aims and methods of the study. It was expressly stated that their participation was voluntary and that they were assured the right to withdraw at any time without facing negative consequences. Ethical approval for the study with reference number (Ref. No. IRB-PGS-2023-04-164) was obtained from the Institutional Review Board (IRB) of Imam Abdulrahman Bin Faisal University (IAU). Compliance with ethical standards was ensured and the protection of the rights and well-being of the participants was a priority. Be. The study strictly adhered to the principles for the inclusion of human subjects set out in the Declaration of Helsinki. To maintain confidentiality, all identifiable information was carefully removed from the data and secure storage was implemented on a dedicated server.

### Data analysis

SmartPLS 3 software was used for data analysis in this study. The analysis included an examination of factor loadings, composite reliability, and mean-variance extracted to ensure convergent validity. Before conducting the structural equation modeling (SEM) analysis, assessments were performed to validate the normality and multicollinearity assumptions. The SEM analysis, conducted using SmartPLS software (version 3.0), aimed to delve deeper into the hypothesized model and examine relationships between latent constructs such as psychosocial, work engagement, and mental health, considering both direct and indirect effects. Bootstrapping was used to generate confidence intervals to determine the significance of relationships within the model. A significance level of *p* < 0.05 was determined with at least 5,000 bootstrap resamples. The results of the SEM analysis were used to evaluate the hypothesized model and examine possible mediating roles of psychosocial, and work engagement. Items that have a low load have been removed.

## Results

### Descriptive statistics

The demographic profile of the study participants provides a comprehensive insight into key characteristics. In terms of gender distribution, 56.5% were female. In terms of age, the majority were in the age group of 30–40 years (51.7%), 23.5% were 41–50 years old and 14.7% were over 50 years old. Marital status revealed that 78.9% were married and 3.5% were either divorced or widowed. Taking nationality diversity into account, 72.5% were Saudi nationals. Educational background varied significantly: 59.2% had a PhD holder and 33.3% had a master’s degree. Teaching experience showed diversity: 55.2% had more than 10 years, 26.4% had 6–10 years, and 18.4% had 1–5 years.

### Measurement model

The reliability and validity analysis presented in Table 1 demonstrates strong psychometric properties for the study constructs. Notably, all items have high loadings, ranging between 0.719 and 0.970, indicating a robust relationship between each item and its corresponding construct. Internal consistency, assessed by Cronbach’s alpha, ranges between 0.595 and 0.933, with rho_A values between 0.742 and 0.946, ensuring reliable measurement across all constructs. Composite reliability values provide additional support for the reliability of the constructs and range from 0.807 to 0.968. The convergent validity, assessed by Average Variance Extracted (AVE), for all constructs, exceeds the recommended threshold of 0.5, which ranges from 0.616 to 0.938. These values mean that each construct captures significant variance relative to measurement error. The Variance Inflation Factor (VIF) values assessing multicollinearity are within acceptable ranges (from 1.22 to 4.27), indicating minimal concerns in this regard. In summary, the analysis shows a measurement model that is characterized by high reliability and validity and is supported by specific numerical values. These robust psychometric results provide a solid foundation for subsequent structural equation modeling analysis and ensure a meaningful investigation of the complex relationships between psychosocial factors, work engagement, and mental health among university faculty in Saudi Arabia.

**Table 1:** Reliability and validity analysis

Construct	Item	Loading	Cronbach’s Alpha	rho_A	Composite reliability	AVE	VIF
Role clarity	RC1	0.929	0.849	0.850	0.930	0.869	2.19
	RC2	0.935					2.19
Work-family conflict	WF1	0.970	0.933	0.935	0.968	0.938	4.27
	WF2	0.967					4.27
Burnout	BO1	0.898	0.740	0.742	0.885	0.793	1.53
	BO2	0.883					1.53
Stress	ST1	0.915	0.727	0.759	0.878	0.783	1.49
	ST2	0.855					1.49
Emotional demand	ED1	0.955	0.595	0.946	0.807	0.682	1.22
	ED2	0.673					1.22
Engagement	ENG1	0.822	0.904	0.907	0.927	0.679	3.06
	ENG2	0.880					4.02
	ENG3	0.863					2.98
	ENG4	0.830					2.35
	ENG5	0.820					2.12
	ENG6	0.719					1.67
mental health	GH2	0.767	0.792	0.795	0.865	0.616	1.56
	GH5	0.820					1.69
	GH7	0.746					1.44
	GH9	0.805					1.70

AVE: Average Variance Extracted

Table 2 the Fornell-Larcker Criterion assesses the discriminant validity of constructs. The diagonal values in the table indicate the square root of the AVE for each construct. The off-diagonal values represent the correlations between different constructs. Discriminant validity is confirmed when the square root of AVE for each construct exceeds the correlation with other constructs. Based on this table, we can generally conclude that discriminant validity is achieved, as the diagonal values are consistently higher than the corresponding off-diagonal values. This suggests that each construct shares more variance with its items than with items from other constructs.

**Table 2:** Fornell-larcker criterion discriminant validity

	Burnout	Emotional demand	Engagement	Mental health	Role clarity	Stress	Work-family conflict
Burnout	***0.891***						
Emotional demand	0.589	***0.826***					
Engagement	-0.492	-0.358	***0.824***				
mental health	0.702	0.519	-0.463	***0.785***			
Role clarity	-0.377	-0.383	0.503	-0.296	***0.932***		
Stress	0.726	0.528	-0.463	0.608	-0.371	***0.885***	
Work-family conflict	0.667	0.474	-0.342	0.620	-0.258	0.511	***0.968***

The bold italicized text is the square root of AVE

Table 3 shows the heterotrait-monotrait correlation ratio (HTMT) for evaluating the discrimination validity of the measurement model. HTMT values close to or below 1 indicate good discriminant validity, with values above 0.85 possibly indicating problems. In this analysis, all HTMT values are below 0.85, confirming that the constructs of burnout, emotional demand, engagement, mental health, role clarity, stress, and work-family conflict are distinct. For example, the HTMT value of 0.773 between burnout and emotional demand suggests appropriate discrimination. Overall, the results confirm the discriminant validity of the measurement model and support the distinctiveness of the study constructs.

**Table 3:** Heterotrait-monotrait ratio of correlations (HTMT) discrimination validity of the measurement model

	Burnout	Emotional demand	Engagement	Mental health	Role clarity	Stress
Emotional demand	0.773					
Engagement	0.598	0.403				
Mental health	0.917	0.651	0.540			
Role clarity	0.476	0.481	0.575	0.359		
Stress	0.976	0.719	0.560	0.785	0.467	
Work-family conflict	0.800	0.553	0.372	0.718	0.290	0.603

Table 4 compares the model fit between the saturated and estimated models. The saturated model shows a better fit compared to the estimated model with lower values for SRMR, d_ULS, and chi-square. While d_G is comparable, the estimated model underperforms slightly in NFI. In conclusion, the saturated model has a better fit according to several indices, highlighting its overall superiority in model fitting.

**Table 4:** Assessment of model fit statistics

	SRMR	d_ULS	d_G	Chi-Square	NFI
Saturated model	0.069	0.988	0.443	1036.162	0.776
Estimated model	0.113	2.703	0.473	969.713	0.791

Table 5 shows R-squared and Q-squared values and provides insights into the explanatory power and predictive relevance of the model for each construct. Notably, burnout has a high R2 of 0.585, indicating that the model explains 58.5% of its variance. Emotional demand, engagement, mental health, stress, and work-family conflict also have varying degrees of explained variance. Furthermore, all Q-squared values are above 0, indicating predictive relevance. In particular, burnout and work-family conflict have high Q-squared valuesof 0.461 and 0.413, respectively, indicating robust predictive ability. Overall, these results demonstrate the effectiveness of the model in explaining and predicting variability in the constructs examined.

**Table 5:** The value of R-square and Q square

	*R* ^2^	Q²
Burnout	0.585	0.461
Emotional demand	0.147	0.089
Engagement	0.266	0.176
mental health	0.551	0.333
Stress	0.138	0.103
Work-family conflict	0.445	0.413

### Structure model

**Fig. 2:**
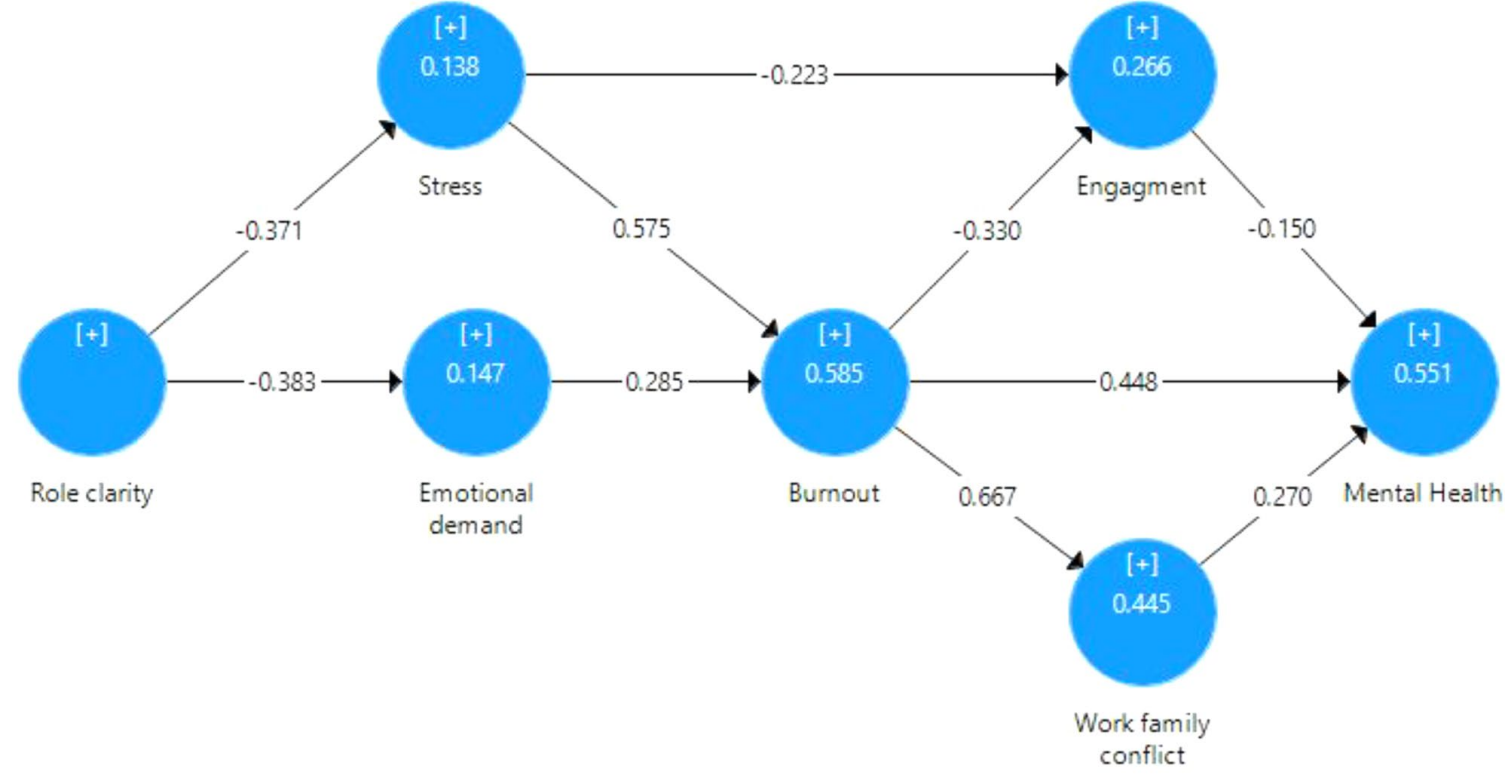
Measurement model

Table 6; Fig. 2 illustrate the path coefficients and results of hypothesis testing for specific paths in the model. Each path coefficient (β) represents the strength and direction of the relationship between variables, with positive and negative values indicating positive and negative relationships, respectively. The t-statistics and highly significant p-values (< 0.001) confirm the statistical significance of these relationships. Notably, the path from burnout to family conflict (H8) and the path from stress to burnout (H4) show significant coefficients with large effect sizes (f2 of 0.800 and 0.576, respectively), highlighting their substantial impact in the model. The categorized effect sizes provide insights into the practical significance of the observed effects. In summary, the outcomes from Table 6 validate the formulated hypotheses and provide a nuanced understanding of the strength and significance of relationships within the analyzed model.

**Table 6:** The path coefficient and hypothesis testing

	Paths	β	SD	T statistics	*P* values	f^2^	Effect size
H1	Role clarity -> Emotional demand	-0.383	0.045	8.438	< 0.001	0.172	Medium
H2	Role clarity -> Stress	-0.371	0.050	7.469	< 0.001	0.160	Medium
H3	Emotional demand -> Burnout	0.285	0.035	8.058	< 0.001	0.141	Small
H4	Stress -> Burnout	0.575	0.033	17.230	< 0.001	0.576	Large
H5	Burnout -> mental health	0.448	0.053	8.428	< 0.001	0.213	Large
H6	Burnout -> Engagement	-0.330	0.069	4.780	< 0.001	0.070	Small
H7	Engagement -> mental health	-0.150	0.043	3.473	< 0.001	0.038	Small
H8	Burnout -> Work-family conflict	0.667	0.029	23.246	< 0.001	0.800	Large
H9	Work-family conflict -> mental health	0.270	0.048	5.606	< 0.001	0.090	Small
H10	Stress -> Engagement	-0.223	0.069	3.221	< 0.001	0.032	Small

Table 7 presents a comprehensive overview of the overall effects of the model, explaining the direct relationships between variables. The total effects (B) illustrate the combined impact of independent variables on dependent variables, with positive or negative values indicating positive or negative associations, respectively. Significant overall effects include the influence of burnout on work-family conflict, burnout on mental health, and stress on mental health, all demonstrated by highly significant p-values (< 0.001) and notable t-statistics.

These results highlight the influential direct relationships within the model and highlight the critical role of burnout and stress in shaping family conflict and overall health. The significance and magnitude of these overall effects provide valuable insights into the complex dynamics within the model under study. Overall, Table 7 improves our understanding of the direct effects and relationships between key variables in the research framework.

**Table 7:** Summary of total effects in the model

	B	SD	T statistics	*P* values
Burnout -> Engagement	-0.330	0.069	4.780	< 0.001
Burnout -> mental health	0.678	0.032	21.412	< 0.001
Burnout -> Work-family conflict	0.667	0.029	23.246	< 0.001
Emotional demand -> Burnout	0.285	0.035	8.058	< 0.001
Emotional demand -> Engagement	-0.094	0.024	3.921	< 0.001
Emotional demand -> mental health	0.193	0.026	7.348	< 0.001
Emotional demand -> Work-family conflict	0.190	0.025	7.548	< 0.001
Engagement -> mental health	-0.150	0.043	3.473	< 0.001
Role clarity -> Burnout	-0.323	0.040	8.095	< 0.001
Role clarity -> Emotional demand	-0.383	0.045	8.438	< 0.001
Role clarity -> Engagement	0.189	0.033	5.657	< 0.001
Role clarity -> mental health	-0.231	0.032	7.265	< 0.001
Role clarity -> Stress	-0.371	0.050	7.469	< 0.001
Role clarity -> Work-family conflict	-0.215	0.030	7.274	< 0.001
Stress -> Burnout	0.575	0.033	17.230	< 0.001
Stress -> Engagement	-0.413	0.045	9.203	< 0.001
Stress -> mental health	0.423	0.030	14.004	< 0.001
Stress -> Work-family conflict	0.384	0.028	13.711	< 0.001
Work-family conflict -> mental health	0.270	0.048	5.606	< 0.001

## Discussion

The objective of the study was to examine the complex relationships between psychosocial factors, work engagement, and mental health within the faculty in Saudi Arabia using structural equation modeling. The result of our study that there is an association between burnout and family conflict is consistent with prior research [23–26]. This result supported the formulated study hypothesis H8 proposing a relationship between burnout and family conflict, and received strong empirical support, aligning with prior research. This result can be explained by faculty who suffer from job burnout becoming dissatisfied with their job and becoming occupied to invest more time and effort after job hours at home to maintain their job. Besides, it is well-documented that burnout causes stress, fatigue, and emotional exhaustion. This leads to failure in meeting family obligations as a partner and parent and therefore results in family-work conflict. It is worth noting, that Saudi Arabia and Arabi culture is based on the strong ties of family relations and insists on the value of extended family connection; therefore, further research in the cultural effect of work-family conflict and job burnout needs more investigation.

This study reveals that university faculty burnout and stress affect their mental health. These findings resonate with existing literature highlighting the influence of burnout on mental health outcomes among faculty [27–29], and nurses [30, 31]. Additionally, research by [32] found a significant connection between burnout and mental health, supporting the significance of these factors in our study. This result proves study hypothesis H4. The empirical evidence thus effectively links the study’s findings to the initially posited hypotheses, reinforcing the theoretical framework. Therefore, we encourage future research to investigate faculty burnout and mental health outcomes through longitudinal and interventional studies to establish such occupational well-being fitness programs to enhance faculty’s health and well-being.

Our study found that faculty’s exposure to psychological risks negatively affects their job engagement. These results are consistent with previous studies [1, 33]. In addition, according to a study conducted by [14], self-perceived health and vigor at work were identified as factors that can predict mental health, which aligns with our research findings. Adding to that, a study conducted by [34] also observed a similar correlation between faculty’s work engagement and compassion fatigue, indicating a negative relationship. On the other hand, our result was inconsistent with a study in the Philippines that revealed no significant differences were identified in the participants’ levels of psychological distress and work engagement [17]. The correlation between psychosocial risks and overall health can be understood through the decline in individuals’ psychosocial well-being, which results in detrimental health outcomes such as psychological and physical symptoms. Consequently, this hampers their participation in activities that promote good health. Therefore, we proposed the implementation of a mental health program as an intervention to alleviate psychological distress and improve work engagement among university faculty.

Additionally, our study’s findings are consistent with those of [3] research, who identified self-perceived health and vigor at work as important predictors of mental health. In contrast, the study conducted by [35] revealed divergent findings, suggesting a notable and adverse association between psychological distress and overall job engagement scores. These differences could be due to variances in the number of participants, the environment in which the study was conducted, and the characteristics of the study group.

This study’s implications are twofold. Firstly, it informs organizational policies and interventions for academic institutions in Saudi Arabia, emphasizing the critical role of addressing burnout to mitigate work-family conflict and enhance overall health among faculty. Secondly, the findings contribute to the broader discourse on faculty well-being globally, highlighting the significance of psychosocial factors and work engagement. Academic institutions worldwide can draw insights to design effective support systems that prioritize mental health. By recognizing the interconnectedness of these variables, institutions can foster a conducive work environment, ultimately improving the overall quality of life and job satisfaction for faculty members in Saudi Arabia and beyond.

Several limitations should be considered in interpreting the study’s findings. Firstly, the cross-sectional design restricts the establishment of causal relationships among variables. Longitudinal studies would provide a more nuanced understanding of the dynamic nature of psychosocial factors, work engagement, and mental health over time. Secondly, the study’s focus on faculty in a specific region of Saudi Arabia may limit the generalizability of results to broader academic contexts. Additionally, self-reported data might introduce response bias, and the reliance on online surveys may exclude individuals with limited internet access. Despite these limitations, the study offers valuable insights into the intricate relationships within the academic setting, paving the way for future research to address these constraints and broaden the scope of inquiry.

## Conclusion

This study explores the relationships between psychosocial factors, work engagement, and mental health among university faculty in Saudi Arabia. The study’s findings underscore the significance of addressing burnout as a central factor influencing work-family conflict and overall health. The identified direct and total effects of key variables contribute valuable insights for organizational interventions and support systems. The study’s outcomes emphasize the need for tailored strategies to enhance faculty well-being, recognizing the intricate interplay of psychosocial dynamics within the academic context in Saudi Arabia.

## Data Availability

Availability of data and materials: The data is available from the corresponding author upon request.

## Abbreviations

COPSOQ: Copenhagen Psychosocial Questionnaire
UWES: Utrecht Work Engagement Scale
GHQ: General Health Questionnaire
SDGs: Sustainable Development Goals
SEM: Structural equation modeling
AVE: Average Variance Extracted
VIF: The Variance Inflation Factor
HTMT: Heterotrait-monotrait correlation ratio
